# Increased expression of deleted in malignant brain tumors (DMBT1) gene in precancerous gastric lesions: Findings from human and animal studies

**DOI:** 10.18632/oncotarget.16792

**Published:** 2017-04-03

**Authors:** Jone Garay, M. Blanca Piazuelo, Lizbeth Lopez-Carrillo, Yelda A Leal, Sumana Majumdar, Li Li, Nataly Cruz-Rodriguez, Silvia J Serrano-Gomez, Carlos S Busso, Barbara G Schneider, Alberto G Delgado, Luis E Bravo, Angela M Crist, Stryder M Meadows, M. Constanza Camargo, Keith T Wilson, Pelayo Correa, Jovanny Zabaleta

**Affiliations:** ^1^ Stanley S. Scott Cancer Center, LSUHSC, New Orleans, LA, USA; ^2^ Division of Gastroenterology, Hepatology, and Nutrition, Department of Medicine, Vanderbilt University Medical Center, Nashville, TN, USA; ^3^ Instituto Nacional de Salud Pública, Cuernavaca, Morelos, Mexico; ^4^ Unidad de Investigación Médica Yucatán de la Unidad Médica de Alta Especialidad (UMAE) del Instituto Mexicano del Seguro Social (IMSS), Yucatán, Mexico; ^5^ Pontificia Universidad Javeriana, Bogotá, Colombia; ^6^ Grupo de Investigacion en Biología del Cáncer, Instituto Nacional de Cancerología, Bogotá, Colombia; ^7^ Department of Otorhinolaryngology, LSUHSC, New Orleans, LA, USA; ^8^ Department of Pathology, Universidad del Valle, Cali, Colombia; ^9^ Department of Cell and Molecular Biology Tulane University, New Orleans LA, USA; ^10^ Division of Cancer Epidemiology and Genetics, National Cancer Institute, Rockville, MD, USA; ^11^ Veterans Affairs Tennessee Valley Healthcare System, Nashville, TN, USA; ^12^ Department of Pediatrics, LSUHSC, New Orleans, LA, USA

**Keywords:** precancerous gastric lesions, *DMBT1*, *H. pylori*, inflammation, gastric cancer

## Abstract

*Helicobacter pylori* infection triggers a cascade of inflammatory stages that may lead to the appearance of non-atrophic gastritis, multifocal atrophic, intestinal metaplasia, dysplasia, and cancer. Deleted in malignant brain tumors 1 (DMBT1) belongs to the group of secreted scavenger receptor cysteine-rich proteins and is considered to be involved in host defense by binding to pathogens. Initial studies showed its deletion and loss of expression in a variety of tumors but the role of this gene in tumor development is not completely understood. Here, we examined the role of *DMBT1* in gastric precancerous lesions in Caucasian, African American and Hispanic individuals as well as in the development of gastric pathology in a mouse model of *H. pylori* infection. We found that in 3 different populations, mucosal *DMBT1* expression was significantly increased (2.5 fold) in individuals with dysplasia compared to multifocal atrophic gastritis without intestinal metaplasia; the increase was also observed in individuals with advanced gastritis and positive *H. pylori* infection. In our animal model, *H. pylori* infection of *Dmbt1−/−* mice resulted in significantly higher levels of gastritis, more extensive mucous metaplasia and reduced *Il33* expression levels in the gastric mucosa compared to *H. pylori*-infected wild type mice. Our data in the animal model suggest that in response to *H. pylori* infection DMBT1 may mediate mucosal protection reducing the risk of developing gastric precancerous lesions. However, the increased expression in human gastric precancerous lesions points to a more complex role of *DMBT1* in gastric carcinogenesis.

## INTRODUCTION

Gastric cancer is the third most frequent cause of cancer deaths in the world [1]. Infection with *Helicobacter pylori* (*H. pylori*) is considered to be an important factor for the development of intestinal-type noncardia gastric cancer [2]. Gastric cancer is the result of a cascade of inflammatory events leading from normal epithelia to non-atrophic gastritis (NAG), multifocal atrophic gastritis without intestinal metaplasia (MAG), intestinal metaplasia (IM), dysplasia and cancer [3–5]. Even though the architecture of each lesion is well known, the molecular mechanisms associated with specific stages are not well characterized. Many factors, including those from the host (i.e. genetic background, gastric juice acidity, diet) and the bacterium (i.e. CagA and VacA virulence factors) may modify the risk of gastric cancer [6, 7]. We have previously shown that variation of single nucleotide polymorphisms (SNPs) in cytokine genes is associated with differential risk of gastric precancerous lesions in African American and Caucasian individuals [8–10]. Some of these SNPs have been also associated with gastric cancer risk in several populations [11–14].

The gene Deleted in malignant brain tumors 1 (*DMBT1*) was first proposed as a candidate tumor suppressor gene because of its inactivation in several medulloblastoma cell lines, as compared with normal cells [15]. *DMBT1* encodes a 340KD glycoprotein containing 14 repeats of the scavenger receptor cysteine-rich (SRCR) domains separated by short serine- and threonine-rich amino acid domains (SIDs), 2 C1r/C1s-Uegf-BMp1 domains, and a carboxy-terminal zona pellucida domain [16, 17]. Thus, DMBT1, also known as Gp-340, muclin, and agglutinin, is composed of motifs that are known to mediate protein-protein interactions and that function as a pattern recognition molecule for Gram-positive and -negative bacteria. In fact, DMBT1 has been shown to bind a wide range of bacteria including *H. pylori*, *Streptococcus mutans, Staphylococcus aureus*, and *Lactobacillus casei*, as well as some viruses [18–22]. It also interacts with other endogenous molecules involved in innate immune response, such as SpA and SpD [16, 23]. As a molecule that participates in host defense, DMBT1 is expressed on various mucosal surfaces such as the gastrointestinal mucosa, the trachea and lungs, and the vaginal mucosa [22, 24–26]. DMBT1 expression is upregulated following infection and/or inflammation [27–31]. DMBT1 may also serve as an epithelial differentiating factor and has been implicated in polarization of epithelial cells [32–34].

Studies on the expression of DMBT1 in the gastric mucosa have reported varied results. Initial reports described DMBT1 expression in normal mucosa as well as in gastric tumors [32, 33, 35]. Kang *et al*. reported induction of *DMBT1* in 100% of intestinal type gastric adenocarcinomas but only in 20% of the diffuse type [32]. Conde *et al*. described upregulation of DMBT1 in 62% of gastric adenocarcinomas [35]). Recent work has shown that DMBT1 expression is practically non-existent in normal gastric mucosa and that is upregulated in areas of intestinal metaplasia and gastric carcinomas as compared to normal tissues [36]. DMBT1 is involved in the modulation of immune responses during inflammatory and infectious processes [37, 38]; however no studies have determined the effect of the lack of this gene in *H. pylori* infection. The goal of the present study was to identify genes associated with the presence of advanced gastric lesions across ethnicities. We report that mucosal *DMBT1* expression is positively correlated with more advanced gastric precancerous lesions in three different ethnic populations; we also show that our mouse model lacking *Dmbt1* (*Dmbt1−/−*) and infected with *H. pylori* develops more severe inflammation and more extensive mucous metaplasia, which is paralleled with increased cell proliferative rates. Genomic analysis of these *H. pylori*-infected Dmbt1 knock-out mice revealed reduced expression of *Il33* mRNA levels and ERK phosphorylation when compared to wild type counterparts. This is interesting because previous research suggest that IL-33 is one of the so called alarmins which initiate tissue recovery after damage or infection (reviewed in [39]). In addition, it has been shown that IL-33 signals through ERK [40, 41]. Our results in mice highlight the role of *DMBT1* in the modulation of the inflammatory cascade and its association with gastric precancerous lesions in response to *H. pylori* infection.

## RESULTS

### Microarray analysis identifies *DMBT1* as a gene associated with advanced precancerous lesions

The goal of this study was to identify genes associated with advanced gastric precancerous lesions. As presented in Supplementary Figure 1A, the scatterplot comparison between MAG and dysplasia samples showed 16 genes with at least 30% change between the two stages (blue dots). Five genes encoding for a transcription factor (*E2F3*), a collagen type I (*COL1A1*), a putative tumor suppressor (*TSC2*), a fibroblast receptor (*FGFR1*) and a sensor of stress-induced growth arrest (*GADD45A*) were overexpressed in MAG. On the other hand, genes genes overexpressed in dysplasia included a protein tyrosine phosphatase (*PTPRH*), a member of the TNF-receptor superfamily (*TNFRS1A*), the retinoic acid receptor alpha (*RARA)*, the pleiotropic cytokine leukemia inhibitory factor (*LIF*), the isoform 1 of the superoxide dismutase (*SOD1)*, the cyclin-dependent kinase 9 (*CDK9*), a member of the lipocalin family (*LCN2)*, a gene encoding for a protein that binds to BCL2 (*BAG1*), a member of the fibroblast growth factor receptor (*FGFR3*), the damage specific DNA binding protein 2 (*DDB2)*, and the deleted in malignant brain tumor (*DMBT1*). Interestingly, only the expression of *DMBT1* was significantly different between the two stages (Table 1). We identified *E2F3* and *DMBT1* as the genes with the largest change in expression when dysplasia samples were compared to MAG (29% reduction for *E2F3* and 2.5 -fold increase for *DMBT1*, Table 1). Using these two genes, we generated heatmaps to determine how well those two genes separated samples from the dysplasia vs MAG stages (Supplementary Figure 1B). Eleven out of 12 dysplasia samples grouped together in one branch of the dendrogram (identified with red arrows), while 29 out of 37 MAG samples grouped together in the other branch. Our results suggest that a significant difference in the expression of *DMBT1* could be used to separate MAG from dysplasia cases.

**Table 1:** Genes with at least 30% fold change expression between dysplasia and MAG

SYMBOL	MAG N=37	Dysplasia N=12	*p*-value	Dysplasia/MAG Ratio
***E2F3***	3263.9±179.5*	2306.9±139.9	0.81495	0.71
***COL1A1***	3068.8±116.9	2272.6±215.5	0.73468	0.74
***TSC2***	4061.5±217.5	3051.6±217.0	0.60963	0.75
***FGFR1***	2805.6±198.6	2139.5±195.7	0.89221	0.76
***GADD45A***	3547.7±354.9	2709±318.2	0.88833	0.76
***PTPRH***	3807.4±263.4	4980.2±511.8	0.87948	1.31
***TNFRSF1A***	3883.7±275.7	5102.1±505.0	0.8205	1.31
***RARA***	2814.2±116.1	3733.4±355.1	0.72687	1.33
***LIF***	2349.9±160.8	3134±280.2	0.6291	1.33
***SOD1***	4413.2±224.9	5901.1±428.9	0.45848	1.34
***CDK9***	2692.9±104.5	3767.4±749.3	0.83884	1.4
***LCN2***	3867.5±267.9	5435.4±704.2	0.77509	1.41
***BAG1***	2603.9±101.2	3700.1±749.3	0.83795	1.42
***FGFR3***	3264.7±220.9	4690.8±647.8	0.74522	1.44
***DDB2***	2333.5±105.8	3392.4±802.5	0.86253	1.45
***DMBT1***	2169.3±174.0	5371.1±665.1	0.00384	2.48

*Mean±SE.

### *DMBT1* expression is higher in gastric precancerous lesions in three different populations

**Figure 1:**
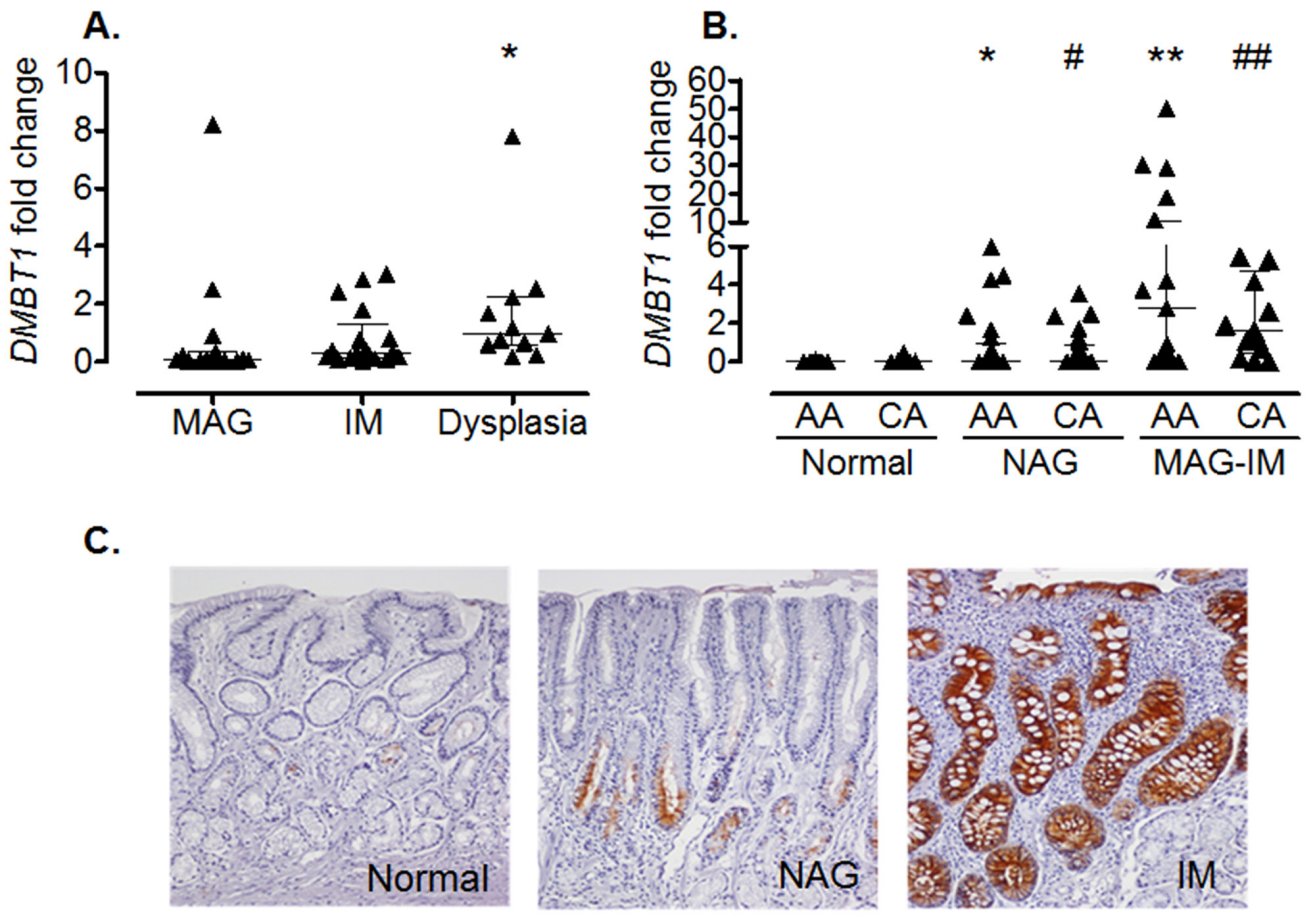
Increased *DMBT1* expression in gastritis and precancerous lesions **(A)** Hispanic individuals with dysplasia have increased levels of *DMBT1* when compared to individuals with MAG (*p=0.0051). **(B)** African American (AA) and Caucasian (CA) individuals with NAG or MAG-IM (combined MAG and IM) show increased gastric levels of *DMBT1* mRNA when compared to individuals with normal gastric mucosa from either ethnic group. *p=0.0058; **p<0.0001 when compared to AA normal; #p=0.0001; ##p<0.001 when compared to CA normal. Median and interquartile range are shown. **(C)** Representative IHC for DMBT1 expression in normal, NAG, and IM.

**Figure 2:**
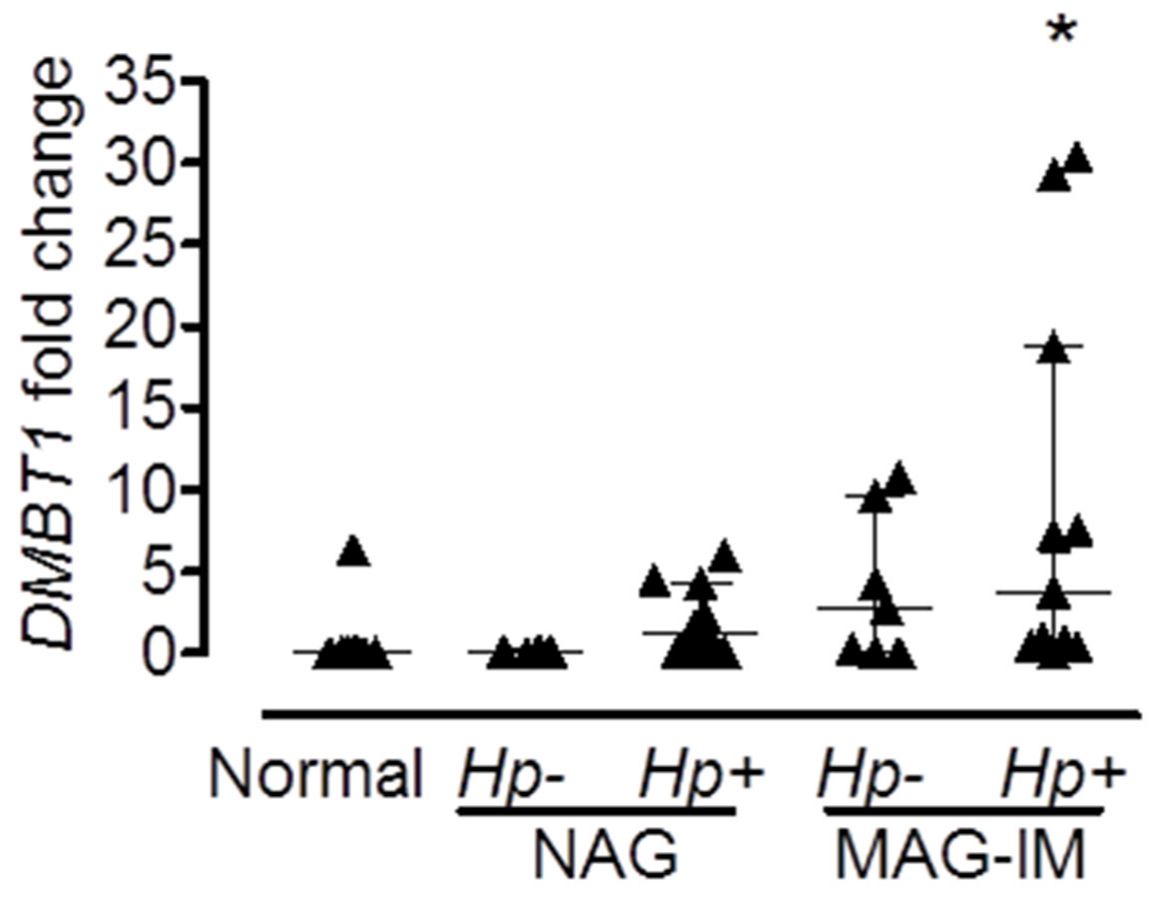
Expression of *DMBT1* is associated with *H. pylori* infection Expression of *DMBT1* from gastric tissue RNA from African American individuals with different stages of gastric lesions was analyzed by real-time PCR. Infection with *H. pylori* was determined by Steiner silver stain as described in Materials and Methods. All subjects with normal gastric mucosa were uninfected. *p=0.007 when compared to Normal; *Hp*, *H. pylori*. Median and interquartile range are shown.

To confirm the results of the microarray analysis and to determine whether the same differences were observed in other populations, we performed real-time PCR in gastric biopsies from Hispanic, African American and Caucasian individuals with various gastric precancerous lesions. Hispanic individuals (Figure 1A) with dysplasia had significantly higher levels of *DMBT1* expression when compared to MAG patients (*p*=0.0051). We also found that *DMBT1* expression was up-regulated in NAG, MAG and IM in both African and Caucasian Americans (Figure 1B), suggesting that this association is conserved across populations. Immunohistochemistry (IHC) staining for DMBT1 showed that African Americans had increased percentage of DMBT1+ epithelial cells in the gastric epithelium when compared with Caucasian individuals (p<0.0001; Table 2). Representative images of DMBT1 IHC are shown in Figure 1C. Since DMBT1 is a known pattern recognition protein, we analyzed its expression according to *H. pylori* infection. As can be seen in Figure 2, African Americans with precancerous gastric lesions (MAG and IM) and infected with *H. pylori* showed the highest *DMBT1* expression, as compared to the other diagnoses (p=0.007 compared to normal mucosa), suggesting an association between the expression of this gene and the infection with *H. pylori*, as has been shown for this protein in other models of infection [42–44]. Taken together, these data show that DMBT1 up-regulation starts very early in the precancerous cascade and increases during the development of the gastric precancerous lesions, suggesting that DMBT1 expression may indicate the transition from inflammatory states to precancerous stages in the cascade of events that lead to cancer.

**Table 2:** DMBT1 epithelial immunostaining in gastric mucosa of African American and Caucasian individuals by diagnostic groups

% of epithelium stained	African Americans			Caucasians		
	Normal n(%)	NAG n(%)	MAG/IM n(%)	Normal n(%)	NAG n(%)	MAG/IM n(%)
0-5	25 (96)	11 (39.3)	7 (50)	24 (96)	19 (67.9)	9 (75)
6-20	1 (4)	11 (39.3)	7 (50)	1 (4)	9 (32.1)	3 (25)
>20	0	6 (21.4)	0	0	0	0

Chi-square 47.83; df=10; p<0.0001. The cut-off point for this analysis was set at 5%.

### Increased inflammatory response in *Dmbt1−/−* mice after *H. pylori* infection

**Figure 3:**
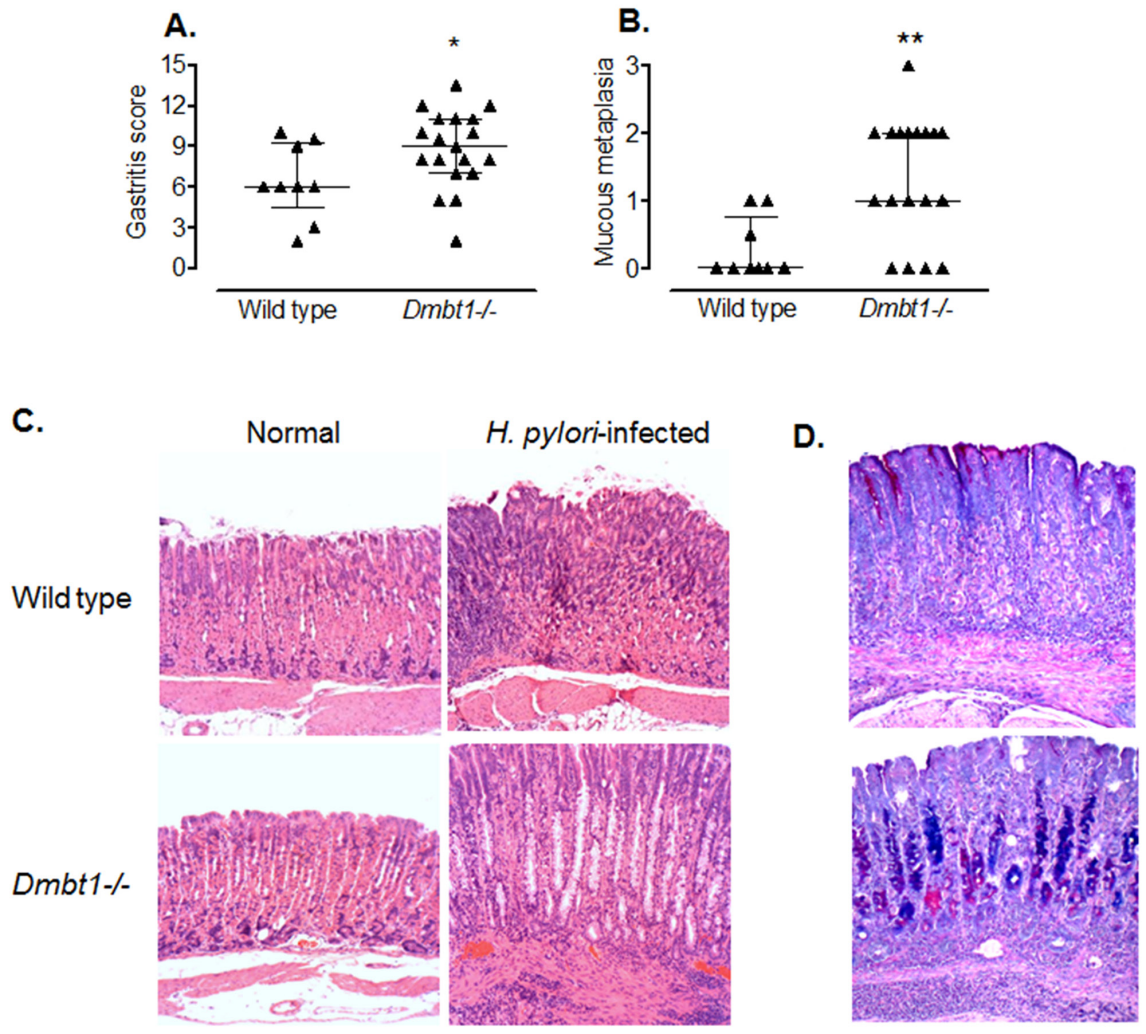
*H. pylori* infection induces premalignant lesions and more severe inflammation in *Dmbt1*−/− mice **(A)** Semiquantitative scores of the inflammatory infiltrate in the gastric mucosae of *Dmbt1−/−* and WT mice 4 months after inoculation with *H. pylori* SS1; *p=0.048. **(B)** Metaplastic changes in the mucosae of the gastric corpus of *H. pylori*-infected *Dmbt1−/−* and WT mice were scored from 0 to 3 as described in Materials and Methods; **p=0.0065. **(C)** Representative images of sections of the gastric corpus of non-infected and *H. pylori*-infected *Dmbt1−/−* and WT mice showing more severe pseudo-pyloric metaplasia in *H. pylori*-infected *Dmbt1−/−* mice compared to *H. pylori*-infected WT mice. H&E stains at 100X magnification. **(D)** Representative images of the AB-PAS stains of gastric mucosa sections of *H. pylori*-infected WT and *Dmbt1−/−* mice showing the presence of mucous metaplasia in *Dmbt1−/−* mice (100X magnification). Median and interquartile range are shown.

We infected WT and *Dmbt1*−/− mice as described in Materials and Methods and determined the level of inflammation and damage in the gastric mucosa. Interestingly, at four months after *H. pylori* infection, the degree of inflammation was already evident and significantly higher in the *H. pylori*-infected *Dmbt1*−/− mice when compared to their WT counterparts (Figure 3A; p=0.048). As previously observed [45], the inflammatory response was higher in the corpus than in the antrum of both infected WT and *Dmbt1*−/− mice. Compared to WT mice, the *Dmbt1-*/- animals also exhibited significantly more atrophy of the oxyntic mucosa with presence of mucous metaplasia (Figure 3B and 3C; p=0.0065). The presence of mucous metaplasia was confirmed by Alcian Blue-Periodic acid-Schiff (AB-PAS) staining, which showed the replacement of fundic parietal and chief cells by foamy cells containing neutral and acid mucins (Figure 3D). As in WT mice, no changes were observed in the gastric mucosa of uninfected *Dmbt1−/−* mice (Figure 3C). Infection of WT mice with *H. pylori* resulted in increased *Dmbt1* mRNA expression; however, *H. pylori* load was not different between *Dmbt1*−/− and WT (Supplementary Figure 2).

**Figure 4:**
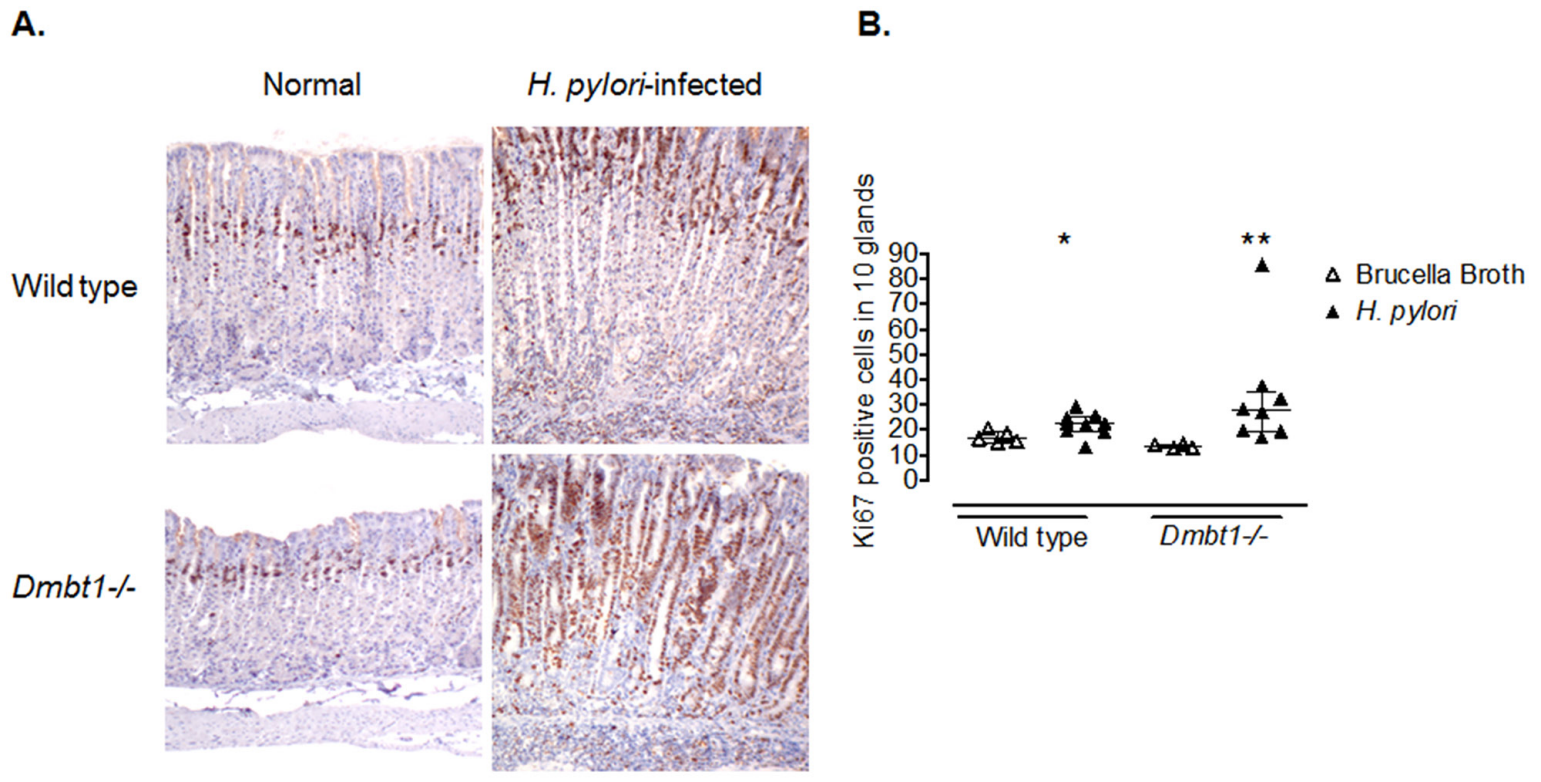
Enhanced proliferation of gastric epithelial cells in *Dmbt1−/−* mice following *H. pylori* infection **(A)** Representative images of Ki67 labelling of gastric mucosa sections of non-infected and *H. pylori*-infected WT and *Dmbt1−/−* mice. **(B)** Ki67-labeledproliferatingcells were counted in 10 well-oriented and randomly selected glands of the gastric corpus; *p=0.042, **p=0.004, compared to the respective non-infected counterpart. Median and interquartile range are shown.

Persistent hyper-proliferation has been suggested to contribute to gastric carcinogenesis [46]. To evaluate gastric epithelial proliferation, we stained sections of the fundic mucosa with Ki67. Although no significant difference in the number of Ki67+ cells was found between *H. pylori*-infected WT and *Dmbt1*−/− mice (p=0.28; Figure 4A and 4B), the infection increased the number of proliferating cells (Ki67+) in both groups of animals. Interestingly, this increase was greater in *Dmbt1*−/− mice (13.6±0.42 in non-infected vs. 33.4±7.9 in *H. pylori*-infected, p=0.004) compared to WT (17.1±1.1 in non-infected vs. 22.2±1.5 in infected animals, p=0.042) (Figure 4B). These results represent a 30% increase in WT compared to 150% in *Dmbt1−/−*.

**Figure 5:**
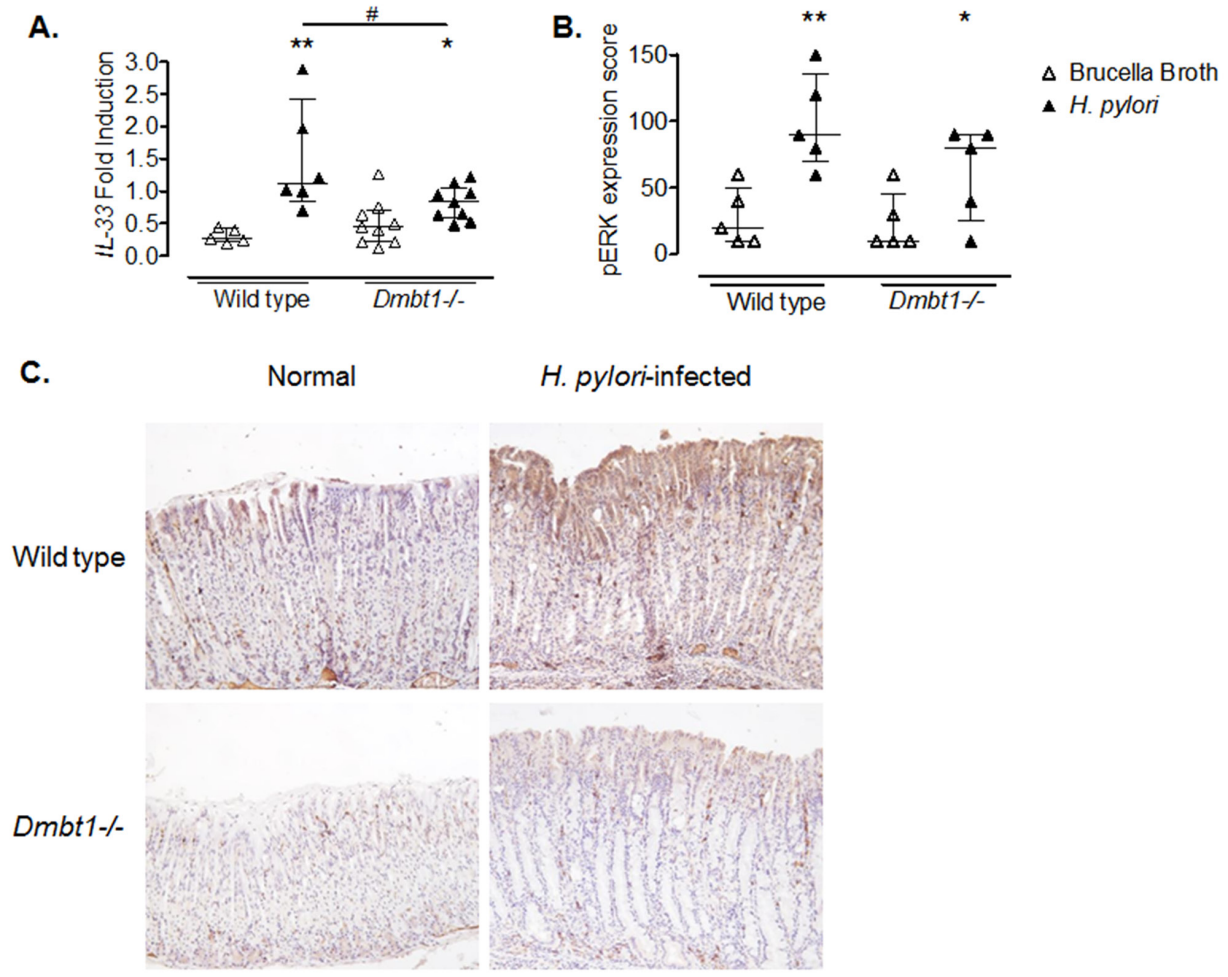
Decreased expression of *IL-33* in *H. pylori* infected *Dmbt1−/−* mice **(A)** Comparison of mRNA levels of *Il33* in the gastric mucosae of *Dmbt1−/−* and WT mice following 4 months of infection with *H. pylori*. Results are shown as fold induction relative to *Gapdh* expression. * p=0.046; **p=0.0043; ^#^p=0.0496. **(B)** pERK expression score was assessed using immunohistochemistry in the gastric mucosae of *Dmbt1−/−* and WT mice infected with *H. pylori* for 4 months as described in Material and Methods. *p=0.095; **p=0.0079. Median and interquartile range are shown. **(C)** Representative images of anti-pERK immunohistochemistry on gastric mucosa sections of non-infected and *H. pylori*-infected WT and *Dmbt1−/−* mice.

To determine whether differences in gene expression could explain the dissimilarities in the inflammatory changes and extension of mucous metaplasia in WT and *Dmbt11*−/− mice in response to *H. pylori* infection, versus their respective controls, we performed microarray analysis looking for genes differentially expressed between infected WT and *Dmbt1−/−* mice. Several genes, including *Il33, Reg1, Muc13* and *Fos*, were downregulated in *H. pylori*-infected *Dmbt1*−/− mice when compared to infected WT mice (Table 3). Interestingly, the gene with the greatest difference in expression between *H. pylori*-infected WT and *Dmbt1*−/− mice was the *Il33* gene. Since IL-33 has recently been shown to have a major role in the immune response following *H*. *pylori* infection [39], we performed real time PCR to confirm our microarray results. We found that WT mice upregulated *Il33* expression in response to *H. pylori* infection up to 350%, while upregulation in the *Dmbt1*−/− reached 52% in response to the infection (Figure 5A). The levels of *Il33* mRNA were significantly higher in *H. pylori*-infected WT than in infected *Dmbt1−/−* mice (p=0.049). As IL-33 signals through ERK, we determined the level of phosphorylation of ERK by immunohistochemistry in WT and *Dmbt1−/−* mice infected with *H. pylori* and found increased levels of of pERK in WT (Figure 5B and 5C).

**Table 3:** Differential gene expression between WT and *Dmbt1−/−* mice in response to *H. pylori* infection

TargetID	WT Signal*	*Dmbt1−/−* Signal	Ratio
***PNLIPRP2***	0.32	0.66	0.48
***H2-EB1***	1.59	1.76	0.90
***CD52***	1.62	1.79	0.91
***C3***	1.76	1.78	0.99
***H2-AB1***	2.01	2.03	0.99
***ZC3H12A***	1.76	1.75	1.01
***MCPT1***	1.61	1.54	1.05
***H2-DMA***	1.98	1.82	1.09
***CDKN1C***	0.46	0.42	1.10
***CD74***	2.44	2.22	1.10
***RPP25***	0.65	0.58	1.12
***H2-AA***	1.98	1.76	1.13
***SLC2A6***	1.92	1.51	1.27
***2010001M09RIK***	4.46	3.34	1.34
***SPRR2F***	3.68	2.66	1.38
***H2-DMB1***	2.60	1.81	1.44
***NFKBIZ***	3.20	2.08	1.54
***CXCL9***	3.18	1.95	1.63
***IIGP2***	3.04	1.78	1.71
***CASP1***	3.68	2.13	1.73
***SMPDL3B***	3.66	2.05	1.79
***H2-K1***	3.25	1.70	1.91
***TYKI***	3.15	1.59	1.98
***DUOXA2***	3.97	2.00	1.99
***VIL1***	3.57	1.54	2.32
***LY6D***	3.61	1.54	2.34
***CD177***	7.55	3.19	2.37
***INDO***	12.29	4.44	2.77
***PSMB9***	5.00	1.72	2.91
***IFIT3***	4.84	1.64	2.95
***SLPI***	4.99	1.67	2.99
***FOS***	4.64	1.53	3.03
***OTTMUSG00000016644***	6.61	2.01	3.29
***MUC13***	14.04	4.16	3.38
***CLCA3***	5.85	1.51	3.87
***REG1***	7.82	1.56	5.01
***IL33***	9.18	1.53	6.00

*Signal was calculated by the ratio between the signal under *H. pylori* infection and non-stimulated conditions.

## DISCUSSION

DMBT1 binds to and aggregates multiple bacterial pathogens including *H. pylori* [18–22]. In some instances, such binding can prevent infection [38, 47]. In addition, a role of DMBT1 in promoting cell differentiation has also been proposed [32, 34, 48].

In the present study, we showed that the expression of DMBT1 is upregulated along the gastric precancerous cascade at both the mRNA and protein level in three different human populations. Furthermore, DMBT1 upregulation occurs as early as the NAG stage in the precancerous cascade, especially in *H. pylori* infected subjects, a finding that suggests a potential association of this gene with *H. pylori* infection in humans. From NAG, the expression of DMBT1 is gradually increased through the consecutive stages of gastric carcinogenesis: MAG, IM and dysplasia. Our results agree with a previous report showing that the expression of DMBT1 is upregulated in intestinal metaplasia compared to normal gastric mucosa [36]. Notably, our work is the first to report a consistent association of DMBT1 with precancerous gastric lesions in 3 different ethnic groups.

To the best of our knowledge, this is the first work that shows the effect of the lack of the gene in a mouse model of *H. pylori* infection. Deletion of *Dmbt1* in mice increased inflammation and exacerbated the gastric *H. pylori*-driven premalignant lesions, as evidenced by greater extent of mucous metaplasia. An increase in proliferative cells was also observed in *Dmbt1−/−* mice. Therefore, taken together, these data suggest that DMBT1 may modulate gastric epithelial damage induced by infection and the inflammatory process and that its deletion promotes disease progression during *H. pylori* infection.

The role of DMBT1 as an innate defense factor has been previously shown in inflammatory diseases. *Dmbt1*-deficient mice for example, have enhanced susceptibility to dextran sulfate sodium (DSS)-induced colitis [31]. Rosenstiel *et al*. reported that DMBT1 inhibits TLR4-mediated NF-κB activation [38]. DMBT1 also has been shown to stimulate the migration of alveolar macrophages [23]. In our study, *Dmbt1−/−* mice had impaired *Il33* response to *H. pylori* infection compared to WT mice. IL-33 is an IL-1 family cytokine that is expressed in barrier tissues, possesses a protective role during infections and promotes a Th2 immune response [39, 49, 50]. Interestingly, IL-33 has recently been reported to be a key player in the immune response to *H. pylori*. Loss of IL-33 after chronic *H. pylori* infection may contribute to the Th1-predominant immune response and epithelial injury that follows [39]. Furthermore, IL-33 has been shown to stimulate regulatory T cells (Tregs) during intestinal inflammation [51]. Tregs are important inflammation suppressors; therefore promoting Treg function is an additional mechanism by which the upregulation of IL-33 may help to limit gastric inflammation and damage to the epithelium. The impaired ability of *Dmbt1*-deficient mice to induce expression of *Il33* in the gastric mucosa may in part explain why infected *Dmbt1−/−* mice had more severe gastric lesions. IL-33 is regulated by trefoil factor 2 (TFF2), a protein involved in mucosal repair [39]. TFF2 is expressed in neck cells of gastric glands and similar to DMBT1, *H. pylori* upregulates TFF2 in gastric tissues. Loss of TFF2 accelerates *H. pylori*-induced gastric premalignant lesions [52]. It has previously been shown that porcine DMBT1 interacts with TFF2 in the stomach, and DMBT1 is therefore considered to be a putative receptor for TFF2 [53]. Even though more studies are required to fully understand the mechanisms involved, our study suggests that DMBT1 is also involved in the regulation of IL-33. IL-33 activates various cell-specific signaling pathways [49, 53]. *In vitro* studies indicate that in human gastric cancer cells, IL-33 signaling occurs through pERK [39]. In agreement with that report, we found significantly higher pERK levels in *H. pylori*-infected WT mice compared to controls. In contrast, *Dmbt1−/−* mice failed to increase pERK after *H. pylori* infection. The latter results suggest that DMBT1 may be involved in the induction of Il33 expression, through phosphorylation of ERK, possibly leading to protective IL-4, IL-5 and IL-10 responses to counteract the inflammatory process in the stomach [39, 49, 54]. It is important to note that we do not know how the mutual expression of *IL33* and *DMBT1* is modulated, if in any way. In addition, even though we found that level of pERK is reduced under *Dmbt1−/−* conditions and seem to be associated with the expression of *IL33*, we acknowledge that this is not be the only way through which *H. pylori* induces the phosphorylation of ERK. The protective effect of DMBT1 in our model of *H. pylori* infection may also be due to the ability of DMBT1 to prevent *H. pylori* from gaining access to the gastric mucosa, as suggested by the fact that degradation of DMBT1 by *E. coli*, for example, increases attachment of the bacteria to epithelial cells [55].

Taken together, our data from the animal model suggest that in response to *H. pylori* infection, *Dmbt1* may be associated with gastric mucosal protection that may reduce the risk of developing advanced gastric precancerous lesions. A major limitation of the mouse model is that wild type mice infected with mouse adapted *H. pylori* strains develop gastritis but do not progress to gastric adenocarcinoma. A possible explanation for that is the fact that the mouse-adapted Sydney strain (SS1), the most widely used mouse adapted *H. pylori* strain and the one used in our study, lacks a functional *cag* pathogenicity island (PAI) [56], a known virulence factor associated with gastric carcinogenesis in humans [57]. In fact, 60% of *H. pylori* strains in the U. S. harbor the cagA PAI, and more than 80% in the Colombian region where part our study was conducted [58]. Unfortunately, infection of mice with *cag*+ *H. pylori* strains frequently results in deletions within the cag PAI [59, 60]. On the other hand, the role of DMBT1 as a protective agent may seem contradictory with our observation of increasing expression of DMBT1 along the human gastric precancerous cascade. Sustained elevation of mucins, glycoproteins closely related to DMBT1, has been shown to promote the transition from chronic inflammation to cancer [61]. DMBT1 may initially increase as a response to *H. pylori* infection and/or the inflammatory response and once upregulated contribute to gastric carcinogenesis. However, the mechanisms by which DMBT1 may contribute to human gastric carcinogenesis are still not fully understood.

## MATERIALS AND METHODS

### Patient populations

For the mucosal expression analysis, we evaluated previously collected formalin-fixed, paraffin-embedded gastric biopsies from Hispanic individuals with preneoplastic lesions who participated in an anti-*H. pylori* eradication trial conducted in Colombia [45, 62, 63]. In particular, a subset of gastric mucosa biopsies at baseline was randomly selected, including 37 with MAG, 25 with IM, and 12 with dysplasia. As a replication set, we evaluated archived gastric biopsies from African American (26 normal, 28 NAG, 21 MAG with or without IM) and Caucasian (28 normal, 29 NAG, 13 MAG with or without IM) individuals attending the gastroenterology services at the Medical Center of Louisiana and the Ochsner Baptist Medical Center (formerly Memorial Medical Center), both in New Orleans, Louisiana, as previously described [8, 10]. All data and specimens were collected under IRB-approved protocols. All subjects provided written informed consent. Gastric mucosa sections were stained with modified Steiner silver stain for evaluation of *H. pylori* infection, as previously reported [64].

### Mice infection

Six-to-eight-week old specific pathogen-free mice with deletion of the *Dmbt1* gene [65] and the corresponding C57Bl/6 controls (The Jackson Laboratories, Bar Harbor, ME) were inoculated by orogastric gavage with 200 μl of Brucella broth containing 10^8^ colony forming units (c.f.u.) of the mouse-adapted Sydney strain (SS1) of *H. pylori*, for 3 consecutive days. Controls were inoculated with Brucella broth alone. Bacteria were grown for 3 days in CDC anaerobic agar plates supplemented with 5% sheep blood (BD Diagnostics, Sparks, MD) under microaerobic conditions using a Campy Pouch system (BD Diagnostics) prior to inoculation. Four or seven months after inoculation, mice were euthanized by CO_2_ inhalation and their stomachs removed, opened along the greater curvature, cut longitudinally, and fixed in 10% neutral-buffered formalin. Mice were housed in specific pathogen-free conditions in the Animal Care Facility at Louisiana State University Health Sciences Center (LSUHSC). Animal handling and experimentation were performed in accordance with the guidelines of LSUHSC- Institutional Animal Care and Use Committee protocols.

### Gene microarray

Analyses of gene expression arrays in humans and mice were done as previously described [45, 66]. Briefly, RNA was extracted from either human gastric tissues fixed in formalin and embedded in paraffin (FFPE) or from fresh frozen gastric tissue from *H. pylori*-infected and uninfected mice using Allprep DNA/RNA FFPE or RNEasy kits, respectively (Qiagen, Valencia, CA). Four hundred ng (400ng) of RNA were used to prepare biotin-labeled cDNA (human) or cRNA (mouse), which was later hybridized to the DASL Gene Expression Assay (human, 502 transcripts) or the mouse WG-6-v2 (30,874 transcripts) chips, respectively, according to the manufacturer's instructions (Illumina Inc, San Diego, CA). The chips were washed and scanned to record the intensity of fluorescence emitted. For data analysis in the GenomeStudio software (Illumina Inc., v2011.1), the samples were normalized as described previously [40] using the detection p value algorithm. Analysis of the human screening array included 37 subjects with MAG, 25 with IM, and 12 with dysplasia. To identify genes associated with advanced gastric precancerous lesions, we compared MAG and dysplasia using MAG as reference, t-test to determine differential expression and scatter plots to identify genes with at least 30% change between the two extreme stages (MAG and dysplasia). Differential expression in mouse microarrays was analyzed separately for wild type (WT) (n=3; non-infected=2) or *Dmbt1*−/− (n=6; non-infected=3) using the uninfected tissue samples as reference and the Illumina Custom algorithm as the error model. Multiple testing corrections using the Benjamini and Hochberg false discovery rate (FDR 0.05) were applied to both analyses. MetaCore software (Thompson Reuters) was used to determine the set of common genes induced in the gastric mucosa after *H. pylori* infection in WT and *Dmbt1*−/− mice (ratio between *H. pylori*-infected and non-infected mice for each strain). Microarray data included in this manuscript have been deposited to the Gene Expression Omnibus (GEO) under accession number GSE83389.

### Real-time PCR

RNA was converted into cDNA using SuperScript III as recommended by the vendor (Life Technologies, Foster City, CA) and subjected to real-time PCR using Assays-on-Demand Taqman probes (Life Technologies, *DBMT1* assay Hs01069306_m1, Mm00455996_m1; *Il33* assay Mm00505403_m1). The fold induction of the genes was determined by the 2^-ΔΔCt^ method using *GAPDH* (Assay Hs03929097_g1; Mm03302249_g1) as a housekeeping gene and an internal control for normalization. Fifteen samples with MAG, 18 with IM and 11 with dysplasia were tested from the Colombian cohort. In addition, samples from African Americans included 26 cases with normal gastric histology, 25 NAG and 21 MAG with and without IM, while tissues from Caucasians comprised 28 normal, 29 NAG and 13 MAG with and without IM.

### Histologic evaluation of mouse gastric mucosa

Paraffin-embedded tissues were cut into 5 μm sections and stained with hematoxylin and eosin for histological evaluation and with modified Steiner silver stain for detection of *H. pylori*. Gastric inflammation was assessed by the addition of the scores for mononuclear and polymorphonuclear infiltrate (0, normal; 1, mild; 2, moderate; 3, severe) and the depth of inflammation (0, normal; 1, limited to mucosa; 2, infiltrating the submucosa) in the antrum and the corpus and then combined to get an overall inflammation score [67, 68]. Mucous metaplasia was scored from 0 to 3, based on the extension of the foamy mucus-containing cells observed in the corpus as previously described [45]. Briefly, the presence of moderate foci of foamy cells affecting less than 1/3 of the parietal cells was scored 1, large foci affecting between 1/3 and 2/3 of the parietal cells was scored 2, and change affecting more than 2/3 of the parietal cells was scored 3. *H. pylori* density was graded from mild to severe (1 to 3).

### Immunohistochemistry

DMBT1 was detected in FFPE human gastric biopsies by using a 1:100 dilution of a mouse monoclonal antibody against human DMBT1 from Santa Cruz Biotechnology (Dallas, TX) and a Mach 2 mouse polymer labeled with horseradish peroxidase (HRP) from Biocare Medical Co. (Concord, CA) as secondary antibody solution (overnight at 4°C and 30 mins at room temperature, respectively). Antigen retrieval was done in citrate buffer in a pressure cooker for 20 minutes and the staining done with DAB for 5 mins at room temperature. The percentage of gastric epithelial cells positive for DMBT1 was determined by light microscopy studying the entire histological sections. Ki67 and pERK detection was performed on mouse gastric tissue in FFPE. Immunohistochemistry (IHC) for Ki67 was conducted using the Mach 2 detection system as described above. A primary monoclonal antibody against Ki67 was used in a 1:100 dilution (BD Pharmingen, San Jose, CA) with overnight incubation at 4°C. Cells positive for Ki67 were counted in 5 to 10 well-oriented glands, and an average positive cell number per gland was calculated. Phosphorylated ERK (pERK) detection was performed using the Avidin-Biotin-Peroxidase complex system, according to the manufacturer's instructions (Vectastain Elite ABC Peroxidase Kit; Vector Laboratories). Antigen retrieval was performed by heating slides in 0.01 M sodium citrate buffer (pH 6.0) to 95°C under vacuum for 40 minutes. Slides were incubated with a monoclonal antibody anti-pERK (SantaCruz, 1:500 dilution) overnight, followed by incubation with biotinylated secondary antibody and then by avidin-biotin peroxidase complex for 1 h at room temperature. pERK immunostaining was evaluated semi-quantitatively assessing the average signal intensity (on a scale of 0 to 3) and the percentage of cells showing a positive stain. Intensity score and percentage of cells stained were then multiplied to give the pERK expression score.

### Statistical analysis

GraphPad PRISM software v4.03 was used to compare all groups by One Way ANOVA and non-parametric tests (Mann-Whitney). The same software was used to determine differences in the percentage of DMBT1+ cells in the gastric mucosa of African American and Caucasian individuals by Chi-square using contingency tables. Heatmaps were created using R software version 3.1.3.

## SUPPLEMENTARY MATERIALS FIGURES


