# Supplementary material for: Increased expression of deleted in malignant brain tumors (DMBT1) gene in precancerous gastric lesions: Findings from human and animal studies
Source: Oncotarget. 2017 Apr 3;8(29):47076–89. doi: 10.18632/oncotarget.16792 (PMC5564545; doi:10.18632/oncotarget.16792)
Supplement: Supplementary file 1 [file oncotarget-08-47076-s001.pdf]

Increased expression of deleted in malignant brain tumors (DMBT1) gene in precancerous gastric lesions: Findings from human and animal studies

SUPPLEMENTARY FIGURES

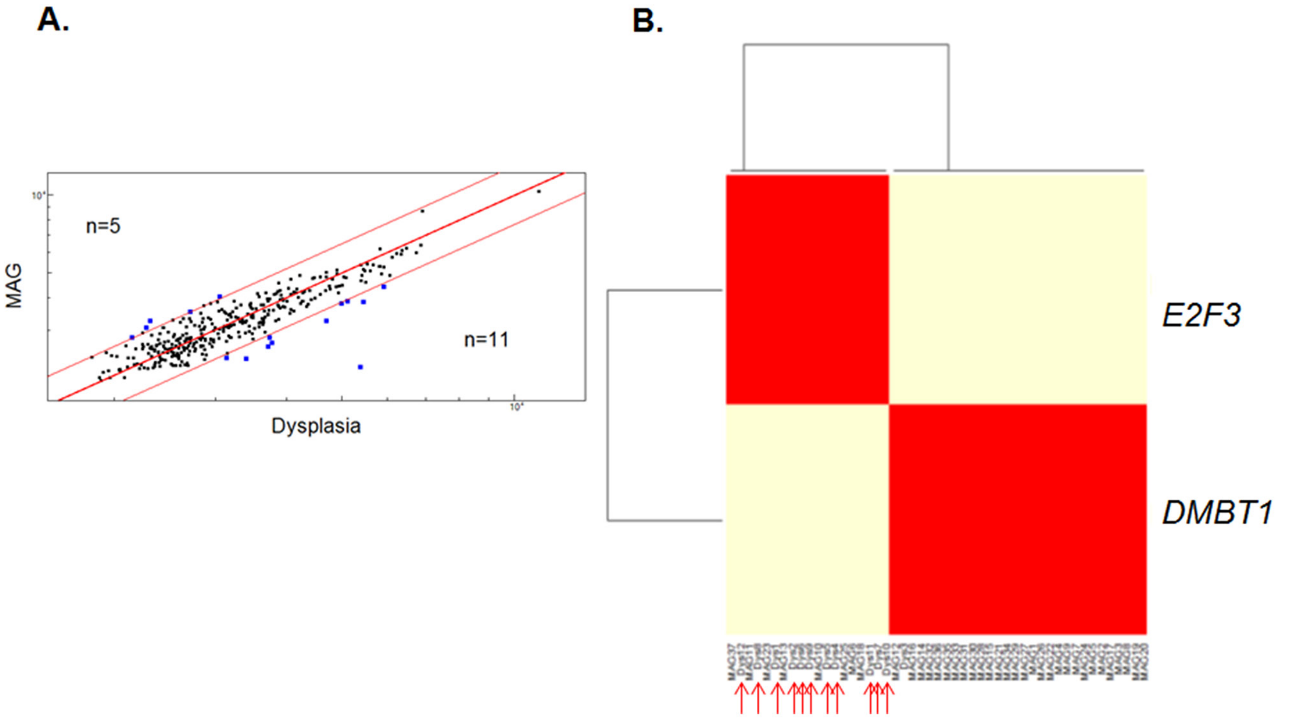

Supplementary Figure 1: Gene differences between MAG and dysplasia. RNA was extracted as described in Materials and Methods. Analysis was done in GenomeStudio. **(A)** Scatterplot analysis showed 16 genes with at least 30% change between MAG and dysplasia stages in Hispanic individuals. **(B)** Heatmap showing separation of MAG and dysplasia (Dys) samples based on the expression of *DMBT1* and *E2F3* genes, the two most modulated genes in advanced gastric lesions. Yellow color indicates higher expression.

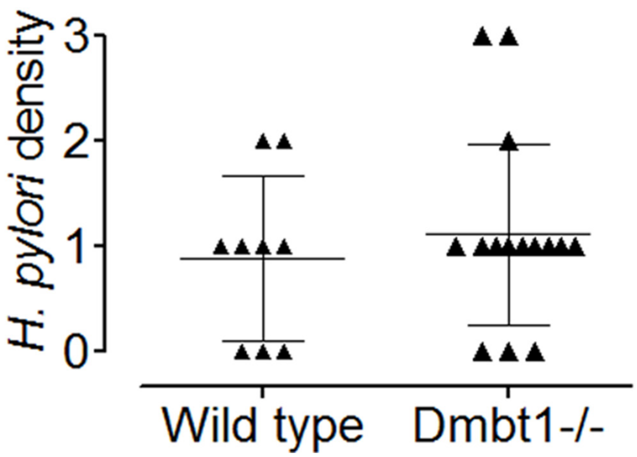

Supplementary Figure 2: *H. pylori* density in WT vs *Dmbt1-/-* mice. Semiquantitative scores of *H. pylori* density in the gastric mucosae of *Dmbt1-/-* and WT mice 4 months after inoculation with *H. pylori* SS1. *H. pylori* density was scored from 0 to 3 as described in Materials and Methods.
